# Protocol to assess calcium signaling in response to metabolic alterations by flow cytometry and live-cell imaging

**DOI:** 10.1016/j.xpro.2026.104435

**Published:** 2026-03-15

**Authors:** Laura Schlautmann, Sven Burgdorf

**Affiliations:** 1 Life and Medical Sciences (LIMES) Institute, University of Bonn, Bonn, Germany

**Keywords:** Flow Cytometry, Immunology, Metabolism, Microscopy

## Abstract

Calcium (Ca^2+^) signaling is essential to cellular processes, such as signal transduction and migration, and dysregulation of Ca^2+^ homeostasis can impact cellular function. Here, we present a protocol to analyze Ca^2+^ signaling by its release into the cytosol in response to and after metabolic stimulation. We describe the steps for cell starvation from excessive nutrients and stimulation, followed by flow cytometry-based and microscopic analysis of calcium signaling. This protocol enables analysis of a large number of cells in a physiologically relevant microenvironment.

For complete details on the use and execution of this protocol, please refer to Schlautmann et al.^1^

## Highlights

• Steps for analyzing calcium signaling by flow cytometry and live-cell imaging
• Detailed instructions on data acquisition and analysis of calcium signaling
• Guidance for determining calcium response to metabolite stimulation

## Abstract (editor highlights)

Publisher’s note: Undertaking any experimental protocol requires adherence to local institutional guidelines for laboratory safety and ethics.

## Graphical abstract

**Figure:**
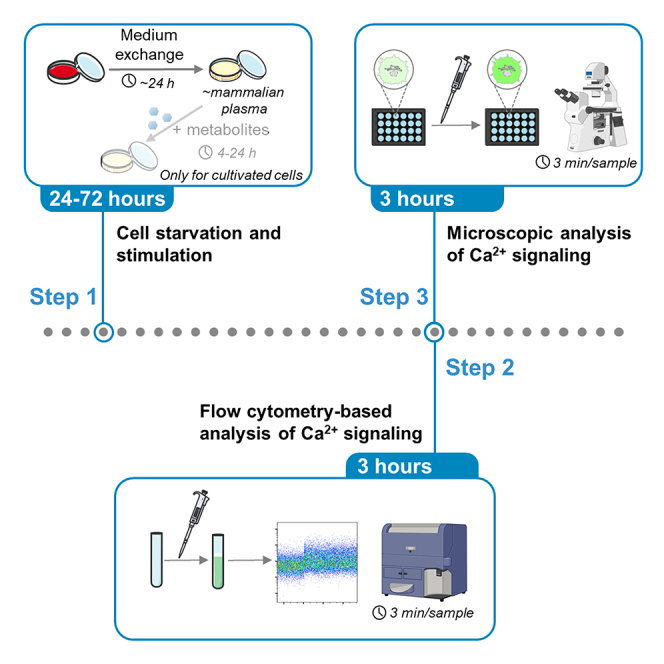

## Before you begin

Calcium (Ca^2+^) is involved as a second messenger in a wide range of signaling pathways crucial for cellular function. In macrophages, the key immune sentinels in our body, Ca^2+^ is essential for various processes, including antigen presentation, phagocytosis, polarization, and cellular migration.^2^^,^^3^ Accordingly, a dysregulation of Ca^2+^ homeostasis can severely influence cellular functions and thus is of crucial importance when exploring cellular function in health and disease.

The incidence of metabolic diseases, including diabetes and obesity, has risen at an alarming rate in recent years. These diseases are characterized by hyperglycemia and have been linked to metaflammation, a chronic, low-grade, systemic inflammation, both of which are driven by macrophages.^4^^,^^5^ Here, we provide a protocol for studying the metabolic influences on Ca^2+^ signaling using flow cytometry as an alternative to live cell imaging, providing an efficient technique for studying kinetic changes in Ca^2+^ signaling in a large number of cells. This protocol is based on cellular loading with the Ca^2+^-sensitive dye Cal-520 AM, which localizes to the cytosol and whose fluorescence is increased upon Ca^2+^ release into the cytosol, either from Ca^2+^ storage compartments such as the ER, or from the extracellular space.

### Innovation

In comparison to conventional techniques to monitor intracellular Ca^2+^ transport, such as fluorescence microscopy, plate reader-based assays or patch clamp analysis, imaging of Ca^2+^ signaling by flow cytometry offers a variety of critical advantages. Most importantly, it allows the rapid analysis of a large number of individual cells with temporal resolution. This easily enables the experimenter to perform and quantify kinetic experiments and to investigate the dynamic influence of specific chemicals or drugs. Additionally, data processing and analysis are straightforward, allowing streamlined examination of the data with high reproducibility. The protocol substantially reduces hands-on time and enables the analysis and comparison of a greater number of conditions due to reduced handling and especially data analysis time. Since for flow cytometry, cell loading with Ca^2+^-sensitive dyes can be performed in bulk, the protocol minimizes variability in dye loading and increases comparability of Ca^2+^ signaling intensities.

Furthermore, cell culture media usually contain excessive non-physiological amounts of nutrients to promote optimal proliferation and sufficient nutrient availability. To resemble a physiologic microenvironment comparable to mammalian plasma, we made use of a cell cultivation protocol using nutrient-deprived media, which creates optimal conditions to study the effect of increased glucose concentrations on cellular function.

### Institutional permissions

Housing and handling of mice, as well as collection and processing of human blood samples, were performed in compliance with national guidelines from Directive 2010/63/EU of the European Parliament and in accordance with the principles of the Declaration of Helsinki, respectively. The investigations have been previously approved by the local institutional animal care and ethics committee.

If the use of primary cells derived from animal or human sources is intended, approval from the respective local and institutional ethics committees is required to ensure compliance.

### Preparation: Cell starvation and stimulation

**Timing: 24–72 h**

The protocol below describes the nutrient deprivation and treatment with metabolic stimuli of cells in culture. This protocol is suited for macrophages from different origins, such as macrophages differentiated from human monocytes or murine bone marrow, or freshly isolated from the murine peritoneum. The protocol can be easily adapted for use of other cell types. In this context it is recommended to check the survival as well as proliferation rate in medium with physiological amounts of nutrients and potentially adapt cell concentrations.

***Note:*** Differentiation of macrophages from murine bone marrow progenitor cells or from human blood monocytes can be induced by the addition of M-CSF or GM-CSF.^1^^,^^6^

***Note:*** If no metabolic treatment is planned before analyzing Ca^2+^ signaling, proceed directly to the step-by-step method details.

***Note:*** For each condition analyzed by flow cytometry 1 to 1.5 million cells are needed. This enables the measurement of Ca^2+^ signaling in response to one stimulus as technical replicate.

1. One day prior to stimulation or use for experiments, deprive cells of excessive nutrients by exchanging the medium to a medium containing physiological levels of glucose (5.5 mM), pyruvate (0.1 mM) and glutamine (0.5 mM).
  a. For adherent cells, replace the supernatant with the according fresh medium (see materials and equipment section).
  b. For non-adherent cells, collect the supernatant, centrifuge at 300 × *g* for 5 min, remove the supernatant and resuspend the cells in the according fresh medium (see materials and equipment section).

***Note:*** For use of cells cultivated in another medium, such as RPMI, use the desired medium with adjusted metabolite concentrations.

***Note:*** If needed, add required growth factors, such as GM-CSF or M-CSF, into the medium.

2. Incubate cells for 24 h at 37°C and 5% CO_2_.
3. Metabolite stimulation.
  a. Stimulate the cells with metabolites and/or potential inhibitors by direct addition into the medium e.g., 11 mM glucose, 1 mM sucralose.

***Note:*** To investigate the instant influence of metabolites on Ca^2+^ signaling, use the cells directly after 24 h of nutrient deprivation and proceed to the step-by-step method details.

4. Incubate for 4 h, 24 h, or 48 h at 37°C and 5% CO_2_.

***Note:*** For highly-proliferating cells, as well as unstable inhibitors, refresh the medium after 24 h of incubation.

### Preparation of dyes and stimuli

**Timing: 30 min**

This section provides information about recommended solvents and stock concentrations for Ca^2+^-sensitive dyes and for the stimuli used to induce Ca^2+^ signaling.

5. To collect the material at the bottom of the vial, spin down vials containing the dyes or stimuli.
6. Dissolve each compound in the appropriate solvent and volume (see Table 1).
7. Prepare aliquots of the stock solution to avoid repeated freeze-thaw cycles.
8. Store aliquots at −20°C.

***Note:*** Protect fluorescent dyes and light-sensitive chemicals from light during handling and store in a dark box to prevent photodegradation or loss of activity.

**Table 1:** Stock solutions for dyes and Ca^2+^-signaling reagents

Compound	Stock concentration	Solvent
2-Deoxy-D-Glucose (2-DG)	555 mM	PBS
Adenosine triphosphate (ATP)	100 mM	Sterile H_2_O
Cal-520 AM	3 mM	DMSO
Cal-590 AM	3 mM	DMSO
Calcium chloride (CaCl_2_)	100 mM	Sterile H_2_O
CCL19	100 μg/mL	Sterile H_2_O
CCL2	10 μM	Sterile H_2_O
Fluo-3 AM	1 mg/mL	DMSO
Fura Red AM	5 mM	DMSO
Ionomycin	1 mg/mL	DMSO
Meso-Erythritol	500 mM	PBS
Sucralose	500 mM	PBS
Thapsigargin	2 mM	DMSO

## Key resources table

**Table:** 

REAGENT or RESOURCE	SOURCE	IDENTIFIER
**Chemicals, peptides, and recombinant proteins**		

DMEM (-D-Glucose, - L-Glutamine, - Sodium Pyruvate)	Gibco	Cat#A1443001
2-Deoxy-D-Glucose (2-DG)	Carl Roth	Cat#CN96.2
Adenosine triphosphate (ATP)	Carl Roth	Cat#HN35.2
Cal-520 AM	AAT Bioquest	Cat#21130
Calcium chloride dihydrate (CaCl_2_), CellPure	Carl Roth	Cat#HN04.2
DPBS, no calcium, no magnesium	Gibco	Cat#14190094
Fetal Bovine Serum (FBS)	PAN-Biotech	Cat#P30-3302
Fluo-3 AM	AAT Bioquest	Cat#21011
Fura Red AM	AAT Bioquest	Cat#21048
Glucose (stock 1.1 M)	Gibco	Cat#A2494001
HBSS, w/Ca^2+^ and Mg^2+^	PAN-Biotech	Cat#P04-32505
HBSS, w/o Ca^2+^ and Mg^2+^	PAN-Biotech	Cat#P04-34500
IMDM (w/5.5 mM Glucose, w/o L-Glutamine)	PAN-Biotech	Cat#P04-20157
Ionomycin calcium salt from *Streptomyces conglobatus*	Sigma-Aldirch	Cat#I0634-1MG
L-Glutamine (stock 200 mM)	PAN-Biotech	Cat#P04-80100
Meso-Erythritol	Thermo Scientific	Car#A15813-14
Penicillin/Streptomycin	PAN-Biotech	Cat#P06-07100
Recombinant murine MCP-1 (CCL2)	ImmunoTools	Cat#12343383
Recombinant murine MIP-3β (CCL19)	PeproTech	Cat#250-27B
Sodium Pyruvate (100 mM)	PAN-Biotech	Cat#P04-43100
Sucralose	Biomol	Cat#Cay31621-25
Thapsigargin	Biomol	Cat#Cay10522-1
UltraPure 0.5M EDTA, pH 8.0	Thermo Fisher	Cat#15575020
β-Mercaptoethanol	Carl Roth	Cat#4227.3

**Experimental models: Cell lines**		

Mouse: J558L GM-CSF producing line	Lutz et al.,^7^, Burgdorf et al.^8^	N/A

**Experimental models: Organisms/strain**		

Mouse: C57BL76 (age: 8–12 weeks, male or female)	Own breeding,	N/A

**Software and algorithms**		

BD FACS Diva	BD Biosciences	N/A
Excel/Word, Office Standard 2019	Microsoft Office	N/A
FlowJo, Version 10	BD Biosciences	N/A
Graphpad Prism, Version 10	GraphPad Software	https://www.graphpad.com/
Image J	Schneider et al.^9^	https://imagej.net/ij/

**Other**		

15 mL/50 mL conical tubes	Sarstedt	Cat#62.554.502
BD LSR II, Flow Cytometer	BD Biosciences	N/A
BD Symphony A5	BD Biosciences	N/A
Cell imaging plate, flat coverglass bottom	Eppendorf	Cat#0030741021
FACS tubes	Sarstedt	Cat#55.1579
Brightfield microscope: Nikon TS1000, Epifluorescence Microscopes: Nikon TI2, Olympus IX81	Nikon, Olympus	N/A

## Materials and equipment

**Table:** Medium containing physiological levels of glucose, pyruvate and glutamine

Reagent	Final concentration	Amount
DMEM (-D-Glucose, - L-Glutamine, - Sodium Pyruvate)	N/A	500 mL
FBS	10%	50 mL
Penicillin/Streptomycin	1%	5 mL
β-Mercaptoethanol (stock 5 mM)	50 μM	5 mL
L-Glutamine (stock 200 mM)	0.5 mM	1.25 mL
Sodium Pyruvate (100 mM)	0.1 mM	0.5 mL
Glucose (stock 1.1 M)	5.5 mM	2.5 mL

Store at +4°C for up to 2 months and warm to 37°C prior to use.

**Table:** 2mM EDTA solution

Reagent	Final concentration	Amount
DPBS	N/A	500 mL
EDTA 0.5M	2 mM	2 mL

Store at +4°C for up to 2 months and warm to 37°C prior to use.

## Step-by-step method details

### Staining with Ca^2+^-sensitive dyes

**Timing: 1.5 h**

In this step, we describe the procedure to load cells with cell-permeable Ca^2+^-sensitive fluorescent dyes.

1. Prepare your desired amount of FACS tubes.
  a. Each condition requires one tube for each compound that will be used for stimulation.
  b. Additionally, include an unstained control.
2. Harvest the cells and transfer to a 15 mL conical tube.
  a. For non-adherent cells: Transfer the supernatant.
  b. For adherent cells: Remove the supernatant. Add 2 mM EDTA solution and incubate at 37°C for 5 min. Detach the cells by flushing and transfer the solution.
    ***Note:*** Also, other methods to detach adherent cells might be used. However, non-enzymatic detachment is preferred to prevent cleavage or inactivation of receptors used for stimulation.
  c. Note the volume.
    ***Note:*** Use cells cultured in nutrient-deprived medium for 24 h or cells additionally stimulated with elevated metabolite concentrations for 4 h to 48 h. Alternatively, use freshly isolated cells, such as murine peritoneal macrophages or PBMCs.
3. Count the cells using a hemocytometer.
4. Centrifuge the cells at 300 × *g* for 5 min. Remove the supernatant.

***Note:*** Depending on your cells, adjust the centrifugation speed to minimize cellular stress while ensuring efficient pelleting of the cells at the bottom of the tube. For human cells, 350 × *g* was used instead of 300 × *g*.

5. Resuspend the cells in warmed HBSS with a concentration of 1.5∗10^6^ cells/mL.

***Note:*** Usually HBSS with (w/, 1.26 mM) Ca^2+^ was used. If you want to prevent cellular Ca^2+^ influx from the cell membrane, perform the experiments in HBSS without (w/o) Ca^2+^.

**CRITICAL:** For every step during the staining procedure, as well as for flow cytometric analysis, warm the HBSS to 37°C prior to experiments and use centrifuges at 22°C. Cooling of the sample will prevent proper cellular responses.

6. Transfer 1 mL into FACS tubes.

***Note:*** If multiple stimuli are planned per condition or if the target population constitutes only a small fraction of all cells, increase the cell number per tube. For multiple stimuli, increase the uptake volume after resuspension; for small target populations, keep the volume to a minimum (as stated in Step 14) to increase the acquisition rate.

7. Add 2 mL warmed HBSS (respectively, w/or w/o Ca^2+^) to each tube.
8. Centrifuge at 300 × *g* (murine cells) or 350 × *g* (human cells) for 5 min.
9. Discard the supernatant by decanting.
10. Load cells with the Ca^2+^-sensitive dye Cal-520 AM.
  a. Prepare 200 μL of staining solution per condition, diluted in HBSS.
    i. The final concentration of the dye should be 5 μM.
      ***Optional:*** Instead of Cal-520 AM, other Ca^2+^-sensitive dyes, such as Fluo-3 AM (1 μg/mL), might be used. It is recommended to perform a microscopic analysis of the cells loaded with the dye simultaneously to ER tracker staining to assess loading properties (as shown in^1^) and select dyes that selectively stain cytosolic Ca^2+^. Table 2 depicts the spectral properties and use of some Ca^2+^-sensitive dyes.
      ***Optional:*** To ensure that observations are not influenced by variable dye loading of single-wavelength Ca^2+^ indicators, validate the results using a ratiometric dye, such as Fura Red (5 μM). Generally, single-wavelength dyes are preferred as these dyes provide higher signal intensities and sensitivity for detecting subtle Ca^2+^ changes.
  b. Add 200 μL staining solution to each FACS tube.
  c. Mix by pipetting.
  d. Incubate for 30 min at 22°C in the dark.
    ***Note:*** To enable cellular uptake, use the dyes in their acetoxymethyl (AM) ester form. Inside the cells, the dye is broken down by esterases, releasing a negatively charged dye that stays inside the cell.
    ***Note:*** If your cells are labeled with GFP or you do not want to use a dye with a fluorescent signal in the FITC channel, use Cal-590 AM.
    ***Optional:*** For the analysis of samples containing various cell types or a heterogeneous mixture of cells, stain cells simultaneously to dye loading with desired antibodies and potentially a LiveDead dye. Also, prepare single-stained controls.
11. Add 2 mL warmed HBSS to each FACS tube.
12. Centrifuge at 300 × *g* (murine cells) or 350 × *g* (human cells) for 5 min.
13. Discard the supernatant by decanting but be careful not to dislodge the pellet.
14. Resuspend the cells in each tube in the desired volume of warmed HBSS (usually 500 μL).

**Table 2:** Setting for flow cytometry for Ca^2+^-sensitive dyes

Compound	Excitation wavelength	Detection filter	Use
Cal-520 AM	Blue laser (488 nm)	525/50	Detection of cytosolic Ca^2+^
Fluo-3 AM	Blue laser (488 nm)	525/50	Detection of total cellular Ca^2+^
Cal-590 AM	Yellow green laser (561 nm)	582/15	Detection of cytosolic Ca^2+^
Fura Red AM	Violet laser (405 nm) Yellow green laser (561 nm)	677/20 670/30	Ratiometric dye to monitor cytosolic Ca^2+^

### Acquisition of calcium signaling by flow cytometry

**Timing: 2–5 min (per sample)**

This section contains the acquisition of the samples at the flow cytometer. Here, all analyses were performed using a BD LSR II or Symphony A5 flow cytometer with the FACS Diva software.

15. Dilute the samples for measurement.
  ***Note:*** It is recommended to measure each condition at least as a technical duplicate.
  a. Mix the cells by pipetting.
  b. Transfer 100 μL of each condition into a new FACS tube.
  c. Add 150 μL of warmed HBSS to each tube.
    ***Note:*** The sample volume for time-based acquisition has been tested for a measurement of up to 3 min at medium speed on a BD LSR II flow cytometer. For other acquisition times or when using different instruments, it is recommended to determine the minimum sample volume needed to sustain acquisition for the desired duration without running out of sample.
    ***Optional:*** To analyze the involvement of enzymes and disruption of cellular signaling cascades, pre-incubate cells with inhibitors for 10 min prior to the analysis of the Ca^2+^ response to your stimulus.
16. Set the voltage at the flow cytometer using the unstained samples.
  a. For panels containing antibodies to gate several cell populations, acquire your single-stained controls.
17. Vortex your cells directly before acquisition.
18. Preparation of experimental setup and global worksheet in FACS Diva.
  a. Prepare a gating strategy within the global worksheet.
    i. Start with a plot (FSC-A × SSC-A) to gate your cells.
    ii. If desired, include additional gating steps for cell populations using your antibody staining.
    iii. Prepare two gates to allow tracking of your fluorescent dye. Plot 1 should be a dot plot with FITC × SSC-A and Plot 2 should display time × FITC.
      ***Note:*** Fluo-3 and Cal-520 were excited with a Blue laser (488 nm) and emission was recorded using a 525 nm filter with 50 nm bandwidth (here termed FITC channel). For Fura Red, signals were analyzed as a ratio of the emission from the Violet laser (excitation: 405 nm) using a 677 nm filter with 20 nm bandwidth (BV650 channel) over the emission from the Yellow green laser (excitation 561 nm) using a 670 nm filter with 30 nm bandwidth (PE-Cy5.5 channel) (see also Table 2).
  b. Define your acquisition parameters.
    i. Set the events to record to a count, which is significantly higher than your expected total count.
    ii. Set the stopping time to the time you plan to measure after the addition of the stimulus (at least 60 s).
      **CRITICAL:** Set your events to record to a too high number to ensure your measurement continues for your planned time period.
      ***Note:*** If you plan to measure for longer time periods, remember to increase the volume in your FACS tube to prevent running out of sample.
19. Acquire your samples.
  a. Vortex your sample and place the sample into the flow cytometer device.
  b. Record a baseline for 40 s.
    i. Press the “Record Data” button in the acquisition dashboard window.
    ii. While measuring, already press the “Stop Acquisition” button. A new window will open named “Confirm”. Additionally, already prepare your stimulus using a microliter pipette.
    iii. After 40 s of recording, press the “OK” button to stop the acquisition and recording simultaneously.
      ***Note:*** Acquire your samples with an acquisition rate of at least 300 events/s. If you are interested in smaller subpopulations, increase your acquisition rate.
      ***Note:*** To ensure stability and sufficient data points for analysis, the baseline recording should last at least 30 s.
  c. Take your sample out of the flow cytometer.
  d. Add your stimulus directly into the cell solution. Ideally, adjust the stock concentration so that you add 10 μL.
    i. Stimulate with a metabolic stimulus at the following concentrations: 2-DG (2.75 to 22 mM), glucose (2.75 to 22 mM), meso-erythritol (5 mM), sodium pyruvate (11 mM), sucralose (0.01 to 5 mM).
    ii. Alternatively, stimulate with extracellular calcium chloride (CaCl_2_, 2 mM), ATP (100 μM), the ionophore ionomycin (1 μg/mL), the SERCA inhibitor thapsigargin (1 μM) or various GPCR ligands, such as CCL2 (14.5 nM) and CCL19 (500 ng/mL).
  e. Vortex the tube and directly place the tube back into the flow cytometer.
  f. Continue recording.
    i. Press the “Record Data” button. A new window (termed “Warning”) will open. Press “Append” to add the data to the baseline measurement.
  g. The recording will stop automatically after your previously set stopping time.
    **CRITICAL:** Exact timing for taking out your sample, adding the stimulus, mixing and placing back is essential to ensure high data quality, continuity and comparability. As well, work as fast as possible to minimize the time delay (typically 2–3 s) after stimulus addition. At the beginning, it is recommended to set a timer to run alongside and ensure consistency in timing between all samples.
20. Export flow cytometry standard (FCS) files.

### Analysis of flow cytometry data

**Timing: ∼1–2 h**

This section describes a basic workflow to analyze the acquired flow data and determine the intensity of the Ca^2+^ signaling in response to the used stimuli to enable comparison between various conditions. Here, the FlowJo Software (BD Biosciences) was used.

**Figure 1:**
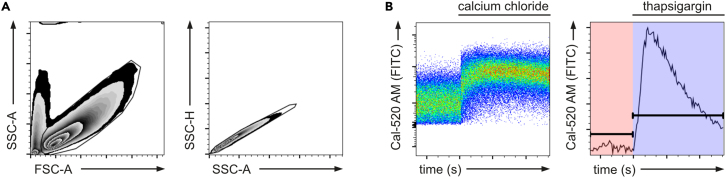
Flow cytometry gating strategy to analyze Ca^2+^ signaling Bone marrow-derived macrophages were stained with the Ca^2+^ indicator Cal-520 AM and analyzed by flow cytometry. (A) Gating strategy to select single, viable cells. Representative plots show exclusion of doublets and debris. (B) Ca^2+^ signaling in response to stimulation with 2 mM calcium chloride (left) or 1 μM thapsigargin in the absence of extracellular Ca^2+^ (right). Representative flow cytometry data after cellular staining with Cal-520 AM, shown as pseudocolor plot (left) and as plot using FlowJo’s kinetics platform (right). Baseline and stimulated gates were defined to enable quantification of Ca^2+^ signaling intensity.

21. Import your FCS files to the analysis software.
22. Gate your single cells using a consecutive gating with an FSC-A × SSC-A and an SSC-H × SSC-A plot (Figure 1A).

***Note:*** If necessary, correct your signals by compensation and gate your populations of interest as described in Schlautmann et al.^1^

23. Analyze the changes in Ca^2+^ levels using the Kinetics Platform (Figure 1B).
  a. Within the “FlowJo” tab, go to “Biology” and then “Kinetics”.
  b. Choose the FITC channel as Y-axis.
  c. Under “Options” – change the statistics parameter to the (geometric) Mean Fluorescence Intensity ((g)MFI).
  d. Add timescale gates for the baseline as well as the stimulated range.

***Note:*** When exporting FCS files from the BD FACS Diva software, the software has an internal error. The time is not output as the added time (sum of time from baseline and after appending), but instead receives the last recorded time range as the time axis. Accordingly, the time resolution is compressed and must be converted for further analysis using a table calculation software.

24. Extract your data for further analyses.
  a. From the Kinetics platform, copy the time series to Excel. This allows to extract a time series with MFI values for every second of the measurement.
  b. Use the table editor to extract information about the gated time ranges.

***Note:*** For further analyses, the statistical parameters AUC (area under the curve) and dt (Delta time, calculated as the difference between end t and start t) of each time range are relevant.

**Figure 2:**
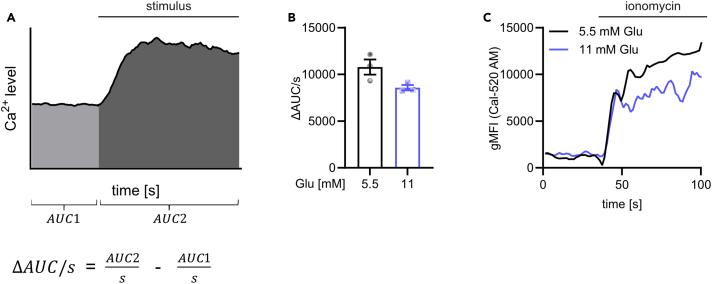
Calculation and visualization of Ca^2+^ signaling intensity Ca^2+^ mobilization in response to ionomycin (1 μg/mL) was monitored by flow cytometry in bone marrow-derived macrophages treated with indicated glucose concentrations for 48 h and stained with Cal-520 AM. (A) Ca^2+^ signaling intensity was determined as difference (Δ) of the Area under the curve (AUC) between the stimulated and baseline region, each normalized to one second. (B) Representative Ca^2+^ signaling intensity calculated as shown in (A) from one exemplary experiment. (C) Ca^2+^ signaling kinetics showing the gMFI over time; Ca^2+^ curve was generated from the same data as depicted in the bar graph (B). Data are presented as mean ± SEM. Glu, glucose; gMFI, geometric Mean Fluorescence Intensity.

25. Data analysis using Excel (or any other spreadsheet program).
  a. Import the time series as well as the kinetics summary statistics into Excel.
  b. Calculate the intensity of Ca^2+^ signaling as the difference (Δ) of the AUC normalized to one second (AUC/s) from the period before and after stimulation (Figure 2A).

***Note:*** It is important to use the same time period and gating for all samples to account for time-dependent signal decrease and enable comparability.

***Note:*** Normalization of the AUC for each phase to 1 s is required to account for differences in phase duration.

***Optional:*** For samples with high variations between experiments, normalize the Ca^2+^ curves. Accordingly, calculate the mean of the control baseline within each experiment and then form the ratio of each value and the respective mean.

26. Depict your data using Graphpad Prism or other graphing software.
  a. Show the Ca^2+^ signaling intensity (ΔAUC/s) as a bar graph with each dot representing the mean of the technical duplicate from one experiment (Figure 2B).
  b. Depict the Ca^2+^ response over time as a pooled curve from each condition over all experiments.
    i. Generate an xy plot with n replicates.
    ii. Visualize the data using the smoothing function (found under “Analyze” – “XY analyses” – “Smooth, differentiate or integrate curve”) by averaging neighboring values (here set to 4) on each size (Figure 2C).

### Validation of calcium signaling response by live-cell imaging

**Timing: ∼1–2 h**

In this section, we provide a protocol to analyze Ca^2+^ levels in response to various stimuli by live cell imaging. This protocol enables tracking of changes in cytosolic Ca^2+^ concentrations within one cell over time. To capture Ca^2+^ signals in the cytosol across the entire cell, epifluorescence microscopy is used.

27. Stimulate and harvest cells as described in step 2.
  a. For non-adherent cells: Transfer the supernatant.
  b. For adherent cells: Remove the supernatant. Add 2 mM EDTA solution and incubate at 37°C for 5 min. Detach the cells by flushing and transfer the supernatant.
  c. Note the volume.
  d. Count the cells using a hemocytometer.
  e. Centrifuge the cells at 300 × *g* for 5 min. Discard the supernatant.
  f. Resuspend the cells in nutrient-deprived medium with 0.5∗10^6^ cells/mL
28. Plate the cells into wells of a glass-bottom plate suitable for microscopy.
  a. Plate 180,000 cells per well into a 24-well glass-bottom plate. Plate at least two wells per stimulus condition.
    ***Note:*** For non-adherent cells, you might need to previously coat the glass surface with Poly-L-Lysine, Collagen or Laminin.
  b. Incubate for 1 h at 37°C or until cells adhere to the plates.
29. Wash the cells with warmed HBSS once cells have adhered to plates.
  a. Remove the supernatant.
  b. Carefully add 500 μL warmed HBSS. Incubate for 30 s and remove the HBSS.
  c. Repeat the previous step.
30. Load the cells with Cal-520 AM.
  a. Add 250 μL of 5 μM Cal-520 AM diluted in HBSS to every well.
    ***Note:*** Adjust the volumes accordingly if you use another well format and ensure that the liquid covers the whole well.
  b. Incubate for 30 min at 37°C in the dark.
31. Wash the cells with warmed HBSS.
  a. Remove the staining solution.
  b. Carefully add 500 μL HBSS and remove it.
  c. Repeat the previous step.
  d. Add 200 μL warmed HBSS to each well.
32. Acquisition of microscopy images
  a. Start your microscope.
    ***Note:*** Imaging was performed using epifluorescence microscopes. A Nikon TI2 equipped with a Plan Apo λ 40× Ph2 DM objective coupled to a Nikon DS-Qi2 camera, and an Olympus IX81-ZDC equipped with a 60× /1.49 NA apochromat oil-immersion objective coupled to a 16-bit EMCCD camera were used. Ca^2+^ signals for Cal-520 were acquired using excitation at 482 nm and emission collected at 525 nm using appropriate filter sets.
    ***Optional:*** For microscopes equipped with a stationary incubation chamber and a heater, preheat the system to 37°C.
  b. Set up the microscope and prepare the measurement as a time series with one image every second for up to 60 or 90 s.
    **CRITICAL:** Minimize photobleaching by using the lowest possible power of the light source and the shortest feasible exposure time during image acquisition. It is recommended to assess bleaching beforehand for untreated cells without the addition of a stimulus. Furthermore, activate the illumination shutter to further reduce the illumination time of the sample.
  c. Prepare a pipette containing 200 μL of warmed HBSS solution with your stimulus (2× concentrated).
    i. Final concentrations of stimulus should be: 1 μg/mL for ionomycin or 22 mM for glucose or as described in Step 18.
  d. Start the measurement.
  e. After 30 s (30 images), cautiously add the 200 μL to the measured well.
    ***Note:*** Pipette carefully with a steady motion and minimal pressure (typically within 1 s) to prevent cells from moving or detaching within your imaging field.
  f. Let the measurement continue until it stops automatically at your set end time.
  g. Export your data.
33. Visualization and Export of time-lapse data using ImageJ.
  a. Open ImageJ and import your data as a hyperstack with split channels.
    i. Import your data per drag and drop.
    ii. A new window ‘*Bio-Formats Import Options’* will open.
    iii. Under stack viewing select *Hyperstack.* Choose the *Split channels* option in the split into separate windows settings.
    iv. Click OK. The picture will be opened with you pre-defined settings.
  b. Change the image display settings to enhance contrast and better visualize signal intensity differences.
    i. Adjust brightness and contrast using the brightest picture. Open the *B&C* window via *Image → Adjust → Brightness/Contrast.* Scroll through your hyperstack and stop at the brightest image. Adjust the display range (Min/Max) and apply it to all images.
    ii. To exclude oversaturation, it is useful to change the lookup table (LUT) to *HiLo* (available via the program main window). Set the brightness and contrast consistently across all images.
    iii. Then, change LUT preferably to a LUT with a color spectrum, such as the *Fire* LUT, to provide clear contrasts between high and low fluorescence regions.
      **CRITICAL:** To allow comparison across conditions, any changes to brightness and contrast must be applied uniformly to all samples.
  c. Export your images as a video (see Methods Video S1).
    i. To preview the movie and adjust the frame rate, e.g., 5 frames per second (fps), go to *Image → Stacks → Animation.*
    ii. Save the stack as a time-lapse video in *AVI* format.
      ***Optional:*** If you want to compare various conditions next to each other within one video, prepare every time series and open them simultaneously in ImageJ. Then, combine the videos next to each other via *Image → Stacks → Tools → Combine*. Afterwards, process the image as described beforehand (see Methods Video S2).
  d. Visualize sequential frames in one panel to compare fluorescence intensity changes over time (as shown in Schlautmann et al.^1^)
    i. Go to *Image → Stacks → Make Montage.*
    ii. Adjust the settings according to your preferences.
    iii. Save the montage image (*File* → Save As → Tiff/PNG).
  e. Export of individual frames from time-lapse stack.
    i. To save a single frame: Scroll to the desired frame. Go to *File → Save as → Tiff* and save the image.
    ii. To save all frames at once in separate files: Go to *File → Save as → Image Sequence.* Choose the file format, name and destination folder.
      ***Optional:*** To determine intensities of Ca^2+^ signaling, extract the fluorescence intensity values of individual cells over time. Therefore, define regions of interest (ROIs) and measure the fluorescence intensity for each time point using the *Multi Measure* option in the ROI manager. Normalize the values to each baseline before addition of the stimulus (mean t = 0–30 s). Calculate the intensity by averaging the mean fluorescence intensity after subtraction of the baseline signal.

Methods Video S1. Representative glucose-induced calcium response, related to step 32 Time-lapse video (5 frames per second) of bone marrow-derived macrophages stained with Cal-520 AM. Cells were stimulated with 22 mM glucose after 5 seconds. Fluorescence intensity is displayed using the Fire lookup table to highlight relative changes in Ca2+ concentrations. Images were acquired every second. Scale bar: 100 μm.

Methods Video S2. Representative ionomycin-induced calcium response, related to step 32 Time-lapse video (5 frames per second) of Ca2+ mobilization in response to 1 μg/mL ionomycin in bone marrow-derived macrophages, treated with physiological (5.5 mM, left) or elevated (11 mM, right) glucose concentrations for 48 h and stained with Cal-520 AM. Ionomycin was added after 30 seconds. Fluorescence intensity is displayed using the Fire lookup table to highlight relative changes in Ca2+ concentrations. Images were acquired every second. Scale bar: 30 μm.

## Expected outcomes

**Figure 3:**
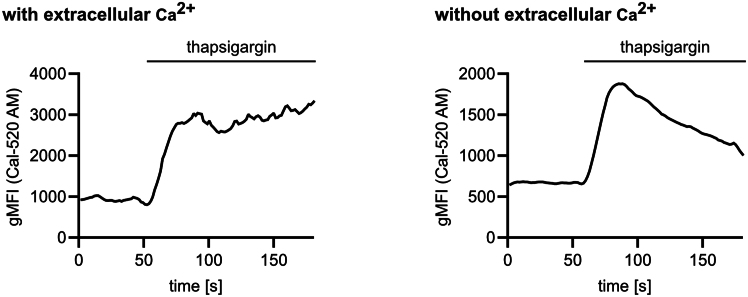
Ca^2+^ signaling dynamics depend on availability of extracellular Ca^2+^ Bone marrow-derived macrophages were stained with Cal-520 AM and Ca^2+^ mobilization to thapsigargin (1 μM) was analyzed by flow cytometry in the presence (left) or absence (right) of extracellular Ca^2+^ (1.26 mM). Representative Ca^2+^ signaling curves from one experiment, measured as technical duplicates (with Ca^2+^) or triplicates (without Ca^2+^).

This protocol enables the detection and quantification of changes in intracellular Ca^2+^ levels by flow cytometry. The protocol allows to measure the rise in intracellular Ca^2+^ levels in macrophages upon addition of metabolic or Ca^2+^ signaling inducing stimuli, such as glucose, thapsigargin or sucralose. In conditions, where extracellular Ca^2+^ is present, elevated Ca^2+^ levels are sustained after stimulation. In contrast, when extracellular Ca^2+^ is absent, the initial rise in cytosolic Ca^2+^ levels is followed by a gradual decline and return to the baseline due to Ca^2+^ back transport into the ER by SERCA (Figure 3).

Flow cytometry analysis produces data with a clear temporal separation between basal and stimulated populations (Figure 1B). Overall, this protocol allows a robust, high-throughput quantification of Ca^2+^ signaling dynamics. Transferring the protocol and staining procedure to other cell types also appears feasible, as a Ca^2+^ response to ionomycin and thapsigargin was also observed in B and T cells gated from human PBMCs.^1^

## Limitations

This protocol enables quantification of Ca^2+^ signaling by high-throughput flow cytometry, allowing analysis of large cell numbers with rapid and straightforward data processing. However, flow cytometric analysis cannot trace individual cells over time and therefore assumes that all cells respond in the same way to a given stimulus. When analyzing heterogenous cell populations, special care should be taken to add specific antibodies and to gate for the individual subpopulations. Alternatively, stable cell lines should be generated to ensure homogenous results. In addition, Ca^2+^-sensitive dyes report relative changes in Ca^2+^ levels rather than absolute ion concentrations. Variability in dye loading efficiency, esterase activity, or cell viability can affect signal intensity and comparability between experiments or cell types.

Due to the temporal resolution of image acquisition (in seconds), the delay introduced by sample handling during mixing and placement in the device, as well as the rapid kinetics of Ca^2+^ signaling in response to strong stimuli, the initial rise in Ca^2+^ influx cannot be captured.

## Troubleshooting

### Problem 1

Cells do not respond to stimuli (related to steps 19 and 32).

### Potential solution

Ensure that all solutions are warmed to 37°C to maintain cellular metabolic activity. If possible, test the Ca^2+^ response with a stimulus known to reliably activate your cell type. Although all cells respond to ionomycin, it is recommended to use a more physiological stimulus, if feasible, with a signal intensity similar to that expected for your tested condition.

### Problem 2

Signal intensity in microscopy images decreases over time-lapse (related to step 32).

### Potential solution

To avoid photobleaching, reduce the power of the light source as well as the exposure time. Additionally, ensure that the shutter closes and the light turns off between the measurements.

### Problem 3

Strong variation in fluorescence signal over time within one measurement (related to steps 15–19).

### Potential solution

Ensure that samples are thoroughly mixed before acquisition to maintain an even cell suspension. Furthermore, verify that the acquisition rate remains stable and sufficiently high (above 300 events/s) and increase the flow rate if the population of interest makes up only a small proportion of your cells. This minimizes the influence of outliers and improves the reliability of the mean fluorescence intensity.

### Problem 4

Inconsistent response to one stimulus between conditions (related to step 15–19).

### Potential solution

Minimize variability by measuring every condition at least in technical duplicates. In addition, measure the first replicate of each condition sequentially for a given stimulus, followed by the second set of replicates. This approach reduces potential effects on the Ca^2+^ response due to delayed acquisition and prolonged incubation in HBSS, which may impact temperature, dye signal intensity (due to dye leakage), and cellular metabolic activity.

## Resource availability

### Lead contact

Further information and requests for resources and reagents should be directed to and will be fulfilled by the lead contact, Sven Burgdorf (burgdorf@uni-bonn.de).

### Technical contact

Technical questions on executing this protocol should be directed to and will be answered by the technical contact, Laura Schlautmann (laura.schlautmann@uni-bonn.de).

### Materials availability

This study did not generate new unique reagents.

### Data and code availability

All data presented in this paper are derived from the same experiments and have been reported in Schlautmann et al.^1^ This study did not generate new codes.

## Acknowledgments

This work was supported by the 10.13039/501100001659Deutsche Forschungsgemeinschaft (10.13039/501100001659German Research Foundation) in the SFB1454 (project 432325352) and in Germany’s Excellence Strategy EXC2151 (project 390873048). The graphical abstract was created using templates from Servier Medical Art (https://smart.servier.com), which are licensed under a CC BY 4.0 (https://creativecommons.org/licenses/by/4.0/).

## Author contributions

L.S. and S.B. conceptualized and planned the experiments. L.S. performed the experiments and analyzed the data. S.B. supervised and funded the project. L.S. and S.B. wrote and revised the manuscript.

## Declaration of interests

The authors declare no competing interests.
